# The role of the North Atlantic Oscillation in controlling U.K. butterfly population size and phenology

**DOI:** 10.1111/j.1365-2311.2012.01359.x

**Published:** 2012-06

**Authors:** Angus R Westgarth-Smith, David B Roy, Martin Scholze, Allan Tucker, John P Sumpter

**Affiliations:** 1 Institute for the Environment, Brunel UniversityUxbridge, U.K; 2 Biological Records Centre, Centre for Ecology & HydrologyWallingford, U.K; 3 Department of Information Systems and Computing, Brunel UniversityUxbridge, U.K

**Keywords:** *Anthocharis cardamines*, *Aphantopus hyperantus*, climate change, *Lasiommata megera*, *Melanargia galathea*, North Atlantic Oscillation, phenology, *Polyommatus icarus*, *Pyronia tithonus*, voltinism

## Abstract

1. The North Atlantic Oscillation (NAO) exerts considerable control on U.K. weather. This study investigates the impact of the NAO on butterfly abundance and phenology using 34 years of data from the U.K. Butterfly Monitoring Scheme (UKBMS).

2. The study uses a multi-species indicator to show that the NAO does not affect overall U.K. butterfly population size. However, the abundance of bivoltine butterfly species, which have longer flight seasons, were found to be more likely to respond positively to the NAO compared with univoltine species, which show little or a negative response.

3. A positive winter NAO index is associated with warmer weather and earlier flight dates for *Anthocharis cardamines* (Lepidoptera: Pieridae), *Melanargia galathea* (Lepidoptera: Nymphalidae), *Aphantopus hyperantus* (Lepidoptera: Nymphalidae), *Pyronia tithonus* (Lepidoptera: Nymphalidae), *Lasiommata megera* (Lepidoptera: Nymphalidae) and *Polyommatus icarus* (Lepidoptera: Lycaenidae). In bivoltine species, the NAO affects the phenology of the first generation, the timing of which indirectly controls the timing of the second generation.

4. The NAO influences the timing of U.K. butterfly flight seasons more strongly than it influences population size.

## Introduction

Climate change is affecting U.K. butterfly populations; the northern distribution limits of some species are moving northwards (Asher *et al.*, 2001, 2011; Hill *et al.*, 2002) and most species are flying earlier (Sparks & Yates, 1997; Roy & Sparks, 2000). It is predicted that projected climate change may cause future population changes (Roy *et al.*, 2001). Insects are excellent organisms through which to investigate the influence of weather as they are poikilothermic and are therefore strongly influenced by climatic conditions. The present study investigates the effect of the North Atlantic Oscillation (NAO) on butterfly ecology, using data from the U.K. Butterfly Monitoring Scheme (2010), which currently contains 16.4 million butterfly records and is one of the best long-term biodiversity datasets in the world.

The NAO exerts considerable control on the weather in the North Atlantic, Mediterranean, Europe and Scandinavia (Hurrell & Deser, 2010). The NAO is described by the NAO index, which is calculated according to air pressures in Iceland and a location within the region of the Azores high pressure area. The NAO exerts a stronger influence on temperature than on precipitation and has its greatest impact on weather in the autumn and winter. A positive NAO index is associated with depression systems taking a more northerly route across the Atlantic, which causes U.K. weather to be milder with slightly higher precipitation, or slightly more maritime in nature; a negative NAO index is associated with depression systems taking a more southerly route, as a result of which U.K. weather tends to be colder and drier, or slightly more continental in nature (Osborn, 2000).

The NAO index is associated with a range of meteorological factors including temperature, precipitation, cloud cover and storms, and so can be a more useful means of describing the variability of the weather experienced by an organism than a single variable such as temperature (Stenseth *et al.*, 2003; Hurrell & Deser, 2010). In effect, the NAO index is a synthesis of a range of weather features that interact to affect organisms. The NAO exerts most of its control on weather before the butterfly flight season (Table 1), which makes it potentially more useful than mean annual temperature to explain butterfly ecology because mean annual temperature is the mean of temperatures over all 12 months, including months that come later in the year after butterflies have finished flying.

**Table 1:** Pearson correlation coefficients (*r*) and probabilities (*P*) between monthly North Atlantic Oscillation (NAO) indices, mean monthly temperatures and monthly precipitation

	Temperature		Precipitation	
		
	*r*	*P*-value	*r*	*P*-value
October	0.446	0.008	0.052	0.769
November	0.327	0.059	0.228	0.195
December	0.649	<0.001	0.226	0.199
January	0.568	<0.001	0.381	0.026
February	0.727	<0.001	0.395	0.021
March	0.367	0.033	−0.068	0.704
April	0.362	0.036	−0.011	0.949
May	0.219	0.213	0.085	0.631
June	0.048	0.790	−0.342	0.048
July	−0.020	0.911	−0.116	0.515
August	0.169	0.341	−0.100	0.574
September	0.193	0.273	−0.324	0.062

September is the last month of the year when butterflies are counted by the U.K. Butterfly Monitoring Scheme and thus the table runs from October of the previous year (dataset for 1975–2008) to September of the current year (dataset for 1976–2009).

A study of the spring arrival time in Finland of 81 migratory bird species showed that most species arrived earlier in years with a positive NAO index, which were therefore characterised by mild, rainy weather. This association was significant for 79% of the species and the correlations were stronger for earlier, rather than later, phases of the migration (Vähätalo *et al.*, 2004). Spring migrant birds have also been found to arrive earlier in years with a positive NAO index in the Czech Republic (Hubalek, 2003) and on Helgoland (Hüppop & Hüppop, 2003). However, the arrival time of trans-Saharan migrant birds to the Mediterranean area can also be influenced by factors including vegetation growth in their overwintering and passage areas and different weather conditions, depending on whether they take a western or an eastern route through the Mediterranean (Robson & Barriocanal, 2011); this indicates that there may be many environmental variables in different geographical regions that influence the phenology of migrant species.

In freshwater habitats, warmer water associated with a positive NAO index results in the earlier emergence of sea trout (*Salmo trutta* L.) fry in the English Lake District (Elliott *et al.*, 2000). Graphs in the paper by Elliott *et al.* (2000) suggest that the NAO is associated with a variation in water temperature of about 3 °C, which is associated with about 5 weeks of variation in emergence date. Mayfly (Ephemeroptera) nymphs in Wales were found to grow faster during positive phases of the NAO, when the water temperature of the streams they inhabit is warmer (Briers *et al.*, 2004).

In the U.K., warm weather associated with a positive NAO index causes the spring migration of the green spruce aphid, *Elatobium abietinum* (Walker) (Homoptera: Aphididae), to start earlier, continue for longer and include more aphids (Westgarth-Smith *et al.*, 2007). There is also preliminary evidence that the NAO influences U.K. butterfly population size (Westgarth-Smith *et al.*, 2005a, b, c).

In marine environments, copepod population size in the eastern North Atlantic and North Sea is affected by the NAO through temperature and wind speed interacting with interspecific competition between two species of copepod (Fromentin & Planque, 1996). Jellyfish (Cnidaria: Scyphozoa) population size in the North Sea appears to be negatively associated with the NAO index (Lynam *et al.*, 2004), although the controlling mechanism is unclear.

The NAO also affects plants; highly significant negative associations have been identified between the NAO index and the leafing dates of 11 tree species and the flowering dates of nine plant species in the U.K. (D’Odorico *et al.*, 2002). The NAO has an impact on U.K. agriculture, as demonstrated by the association between the NAO index and the quality and economic value of wheat (*Triticum* spp.) (Kettlewell *et al.*, 1999).

The aims of the present study were to investigate whether the NAO influences butterfly abundance and phenology, and whether there is an interaction with life history variables, including the number of generations and duration of flight season. The study sought to establish whether it is possible to identify a mechanism whereby weather associated with the NAO in specific months influences butterfly phenology and, if so, whether the mechanism differs for univoltine (one generation per year) and bivoltine (two generations per year) species.

## Materials and methods

### Meteorological datasets

Monthly NAO indices were obtained from the Climate Research Unit (2004) and Osborn (2010). This NAO index uses air pressure data from Iceland and Gibraltar. A winter NAO index was calculated as a mean of the January, February and March NAO indices. Monthly mean temperature data were obtained from the Central England Temperature Series (Parker *et al.*, 1992; Met Office Hadley Centre observation datasets, 2009) and monthly precipitation data were from England and Wales Precipitation (Alexander & Jones, 2001; Met Office Hadley Centre observation datasets HadUKP, 2010).

### The U.K. Butterfly Monitoring Scheme

The U.K. Butterfly Monitoring Scheme (UKBMS) was piloted in Monks Wood in Cambridgeshire, U.K., during 1973–1975, and was then extended nationally from 1976 to include a steadily expanding number of survey sites in the U.K. The monitoring technique involves walking a standardised line transect on a weekly basis from the start of April to the end of September when weather conditions are suitable for butterfly activity. All butterflies seen by the observer in a strip 5 m wide are identified and counted (Pollard & Yates, 1993; U.K. Butterfly Monitoring Scheme, 2010).

Butterfly data for 1976–2009 are available as a multi-species annual collated index, calculated from U.K. abundance data for 49 species (Brereton *et al.*, 2011) and annual collated indices for each species. These collated indices are calculated from all UKBMS sites in the U.K. and represent a national annual index of abundance. Weekly butterfly counts are also available for each UKBMS transect site (U.K. Butterfly Monitoring Scheme, 2010). The peak flight week is the week in the national dataset during which the greatest number of butterflies is seen.

Butterfly life history information, including the typical number of generations per year and the usual months when adults fly, was obtained from Pollard and Yates (1993).

### Choice of species

Although species-level annual collated indices were available for most U.K. species (*n* = 57), those species for which data for the entire time period of 1976–2009 were unavailable were excluded from the present study. In addition, the three main migrants [*Colias croceus* (Geoffroy) (Lepidoptera: Pieridae), *Vanessa atalanta* (L.) and *Vanessa cardui* (L.) (Lepidoptera: Nymphalidae)] were excluded as these species spend part of their lifecycles outside the U.K. and therefore in different meteorological conditions. Therefore, annual collated indices for 35 species were used in the current study. Of these 35 species, 23 species are univoltine and 12 are bivoltine. Bivoltine species may be more strongly affected by the NAO as their two generations are spread over a longer period of the year than the single generation of univoltine species and so are more active at times of the year when the NAO affects the weather.

None of the 35 species had precisely defined peak flight weeks in all 34 years between 1976 and 2009. It can, for example, be difficult to precisely identify a peak flight week if similar numbers of butterflies are counted in each of two consecutive weeks. However, abundant univoltine species with shorter flight seasons generated more accurately defined peak flight weeks in more years than species that were bivoltine, less abundant or had a longer flight season. Accurate determination of the peak flight week for some bivoltine species was reduced if one generation was quite small, or by the presence, in some years, of a third generation. Voltinism is also affected by latitude and thus a species can be bivoltine in southern Britain and univoltine further north. Thus, high-quality datasets suitable for analysis of flight timing are available for only a rather limited number of butterfly species, of which most are univoltine and only two are bivoltine.

Six butterfly species were chosen to investigate the association between the NAO and peak flight week. Data for these species were available and of sufficiently high quality throughout the entire time period studied. These included four univoltine species: *Anthocharis cardamines* (Lepidoptera: Pieridae) (orange tip); *Melanargia galathea* (Lepidoptera: Nymphalidae) (marbled white); *Aphantopus hyperantus* (Lepidoptera: Nymphalidae) (ringlet); and *Pyronia tithonus* (Lepidoptera: Nymphalidae) (gatekeeper or hedge brown). The other two species were bivoltine: *Lasiommata megera* (Lepidoptera: Nymphalidae) (wall brown); and *Polyommatus icarus* (Lepidoptera: Lycaenidae) (common blue).

**Fig. 1:**
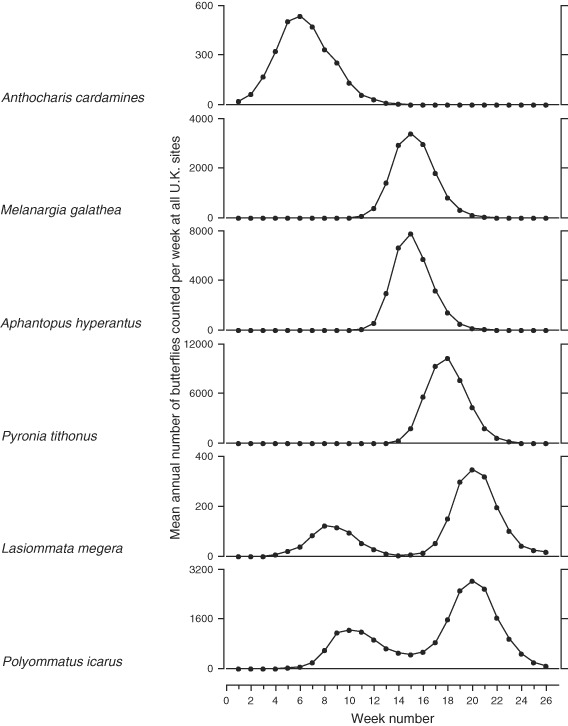
Flight seasons of four univoltine butterfly species, *Anthocharis cardamines*, *Melanargia galathea*, *Aphantopus hyperantus* and *Pyronia tithonus*, and two bivoltine species, *Lasiommata megera* and *Polyommatus icarus*. Data show the annual mean number of each species counted per week at all U.K. Butterfly Monitoring Scheme (UKBMS) sites for 1976–2009. The week numbers are those used by the UKBMS and thus week 1 is the first week of April and week 14 is the first week of July.

The four univoltine species cover two seasons of the year. *Anthocharis cardamines* flies in spring, when the NAO has maximum control over the weather, but not as early as might allow many butterflies to be missed because they are flying before the survey starts or because the weather is too unstable to calculate an accurate peak flight week. *Melanargia galathea*, *A. hyperantus* and *P. tithonus* fly in the summer. The first generations of *L. megera* and *P. icarus* fly in late spring and the second generations in summer (Fig. 1). Therefore, the six species peak at different times and hence should provide an indication of how the NAO affects butterfly populations throughout the spring and summer.

### Statistical analysis of data

Pearson correlation coefficients (*r*) and probabilities (*P*) were used. Percentage control of variability was calculated using a coefficient of determination (*r*^2^) multiplied by 100. Multiple linear regression analysis was used to identify which month's weather had the most influence on peak flight week; regression coefficients (*b*), probabilities (*P*) and overall model coefficients of determination (*r*^2^) were calculated. Variance inflation factors (VIFs) were used as a test for collinearity and a VIF of <5.0 was considered to be free from collinearity. Binary logistic regression coefficients (*β*) were calculated when using the number of generations, as this was a discontinuous variable.

Associations between the winter NAO index and the first and second generation peak flight week numbers were investigated by path analysis. Here we considered two models with the following direct and indirect dependencies: (i) second generation peak flight week numbers are connected only indirectly to the winter NAO index (winter NAO index → first generation → second generation); and (ii) second generation peak flight week numbers are connected directly and indirectly to the winter NAO index (winter NAO index → first generation → second generation, and winter NAO index → second generation).

**Fig. 2:**
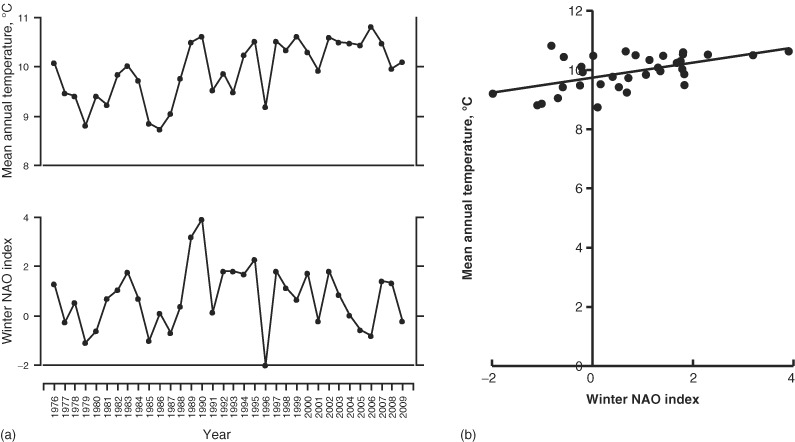
(a) Mean annual temperatures and winter North Atlantic Oscillation (NAO) indices during 1976–2009. Mean annual temperature increased by 1.22 °C (*r* = 0.620, *P*≤ 0.001) during 1976–2009. (b) Association between mean annual temperature and winter NAO index (*r* = 0.544, *P* = 0.001).

Pearson correlation coefficients, multiple linear regression analysis and binary regression analysis was conducted using spss Version 15 (SPSS, Inc., Chicago, Illinois). Coefficients of determination were calculated using Excel (Microsoft Corp., Redmond, Washington). The statistical software C^2^ (Juggins, 2007, 2010) was used to construct the multi-proxy graphs (Figs 1, 2a). Statistical path analysis was performed using the sas procedure proc tcalis (SAS Institute, Inc., Cary, North Carolina).

## Results

### The NAO, climate change and weather

During the period 1976–2009, mean annual temperature recorded in the Central England Temperature Series increased by 1.22 °C. Although the winter NAO index fluctuated considerably during this period, it showed no overall increase or decrease (Fig. 2a).

There was a highly significant Pearson correlation between the winter NAO index and mean annual temperature (*r* = 0.544, *P* = 0.001) (Fig. 2b). To identify the months in which the NAO affects the weather in the U.K., Pearson correlation coefficients were calculated between monthly NAO indices, mean monthly temperatures and monthly precipitation. There were highly significant positive Pearson correlation coefficients (*P* = 0.001) between monthly NAO indices and mean monthly temperatures during December–February and significant positive correlations (*P*≤ 0.05) in October, March and April (Table 1). The correlation for November was not quite significant (*P* = 0.059). The association between the NAO and precipitation was weaker, although there were significant positive correlations between monthly NAO indices and monthly precipitation in January (*P* = 0.026) and February (*P* = 0.021) (Table 1).

### The relationship between the NAO and butterfly abundance

The multi-species collated index was used to assess the association between the collective abundance of many species and the winter NAO index. There was no significant association between the winter NAO index and the multi-species annual collated index (*r* = 0.137, *P* = 0.439, *r*^2^ = 0.0189), which suggests that the NAO does not affect the total population size of all butterfly species in the U.K.

Pearson correlation coefficients were calculated between the annual collated indices for all 35 individual species and the winter NAO index in order to determine if there were associations for individual species. Only one species, *L. megera*, showed a significant positive correlation (*r* = 0.348, *P* = 0.043). However, bivoltine species (*β* = 10.346, *P* = 0.006) and those with longer flight periods (*r* = 0.248, *P* = 0.151) tended to show a stronger positive correlation between the population index and the winter NAO index (Table 2).

**Table 2:** Thirty-five species of butterfly ranked by the Pearson correlation coefficient (*r*) between their respective annual collated indices and the winter North Atlantic Oscillation index

					Months when flying												
					
Species	Common name	*r*	*P*-value	Generations, *n*	J	F	M	A	M	J	J	A	S	O	N	D	Total months
*Lasiommata megera* (L.)	Wall Brown	0.348	0.043	2					•	•	•	•					4
*Anthocharis cardamines* (L.)	Orange Tip	0.310	0.074	1				•	•	•							3
*Lycaena phlaeas* (L.)	Small Copper	0.304	0.080	2					•	•	•	•					4
*Pararge aegeria* (L.)	Speckled Wood	0.277	0.113	2				•	•	•	•	•	•	•			7
*Celastrina argiolu*s (L.)	Holly Blue	0.257	0.142	2				•	•	•	•	•					5
*Polyommatus icarus* (Rott.)	Common Blue	0.237	0.178	2					•	•	•	•					4
*Pieris rapae* (L.)	Small White	0.235	0.181	2					•	•	•	•					4
*Pieris napi* (L.)	Green-veined White	0.185	0.296	2					•	•	•	•					4
*Aphantopus hyperantus* (L.)	Ringlet	0.135	0.447	1							•	•					2
*Hipparchia semele* (L.)	Grayling	0.125	0.482	1							•	•					2
*Erynnis tages* (L.)	Dingy Skipper	0.111	0.532	1					•	•							2
*Coenonympha pamphilus* (L.)	Small Heath	0.106	0.552	2					•	•	•	•	•				5
*Ochlodes sylvanus* (Esper)	Large Skipper	0.103	0.563	1						•	•						2
*Boloria selene* (D. & S.)	Small Pearl-bordered Fritillary	0.102	0.566	1					•	•	•						3
*Maniola jurtina* (L.)	Meadow Brown	0.101	0.570	1						•	•	•	•				4
*Thymelicus sylvestris* (Poda.)	Small Skipper	0.101	0.571	1						•	•	•					3
*Neozephyrus quercus* (L.)	Purple Hairstreak	0.085	0.631	1							•	•					2
*Aglais urticae* (L.)	Small Tortoiseshell	0.083	0.639	2				•	•	•	•	•	•	•			7
*Pieris brassicae* (L.)	Large White	0.058	0.743	2					•	•	•	•					4
*Polygonia c-album* (L.)	Comma	0.035	0.844	2				•	•	•	•	•	•	•			7
*Pyronia tithonus* (L.)	Gatekeeper or Hedge Brown	0.017	0.925	1							•	•					2
*Satyrium w-album* (Knoch)	White-letter Hairstreak	0.008	0.963	1							•	•					2
*Inachis io* (L.)	Peacock	0.003	0.987	1					•	•	•	•					4
*Callophrys rubi* (L.)	Green Hairstreak	−0.017	0.924	1					•	•							2
*Boloria euphrosyne* (L.)	Pearl-bordered Fritillary	−0.023	0.895	1					•	•							2
*Pyrgus malvae* (L.)	Grizzled Skipper	−0.033	0.851	1					•	•							2
*Aricia agestis* (D. & S.)	Brown Argus	−0.057	0.750	2					•	•	•	•					4
*Melanargia galathea* (L.)	Marbled White	−0.079	0.658	1							•	•					2
*Leptidea sinapis* (L.)	Wood White	−0.106	0.553	1					•	•							2
*Argynnis aglaja* (L.)	Dark Green Fritillary	−0.157	0.374	1						•	•	•					3
*Polyommatus coridon* (Poda.)	Chalk-hill Blue	−0.163	0.356	1							•	•	•				3
*Limentis camilla* (L.)	White Admiral	−0.172	0.332	1						•	•	•					3
*Argynnis paphia* (L.)	Silver-washed Fritillary	−0.221	0.209	1						•	•	•					3
*Thymelicus lineola* (Ochsenheimer)	Essex Skipper	−0.235	0.181	1							•	•					2
*Gonepteryx rhamni* (L.)	Brimstone	−0.250	0.154	1				•	•	•	•	•	•				6

The table includes the typical number of generations per year and the duration of the flight season in months. Species with a more positive association with the winter North Atlantic Oscillation index were more likely to be bivoltine (*β* = 10.346, *P* = 0.006) and have a longer flight period (*r* = 0.248, *P* = 0.151).

### The relationship between the NAO and butterfly phenology

The analysis so far has concentrated on the effect of the winter NAO on butterfly abundance, based on collated indices. However, the NAO may also affect the timing, or phenology, of butterfly flight periods.

Six species were chosen in order to investigate the effects of the NAO on phenology. Sample sizes for all of these were large. Samples of *An. cardamines* ranged from 348 individuals counted in 1976 to 7715 counted in 2009; the number of sites ranged from 24 in 1976 to 617 in 2009. Samples of *M. galathea* ranged from 694 individuals in 1978 to 48 092 in 2006; the number of sites ranged from 11 in 1976 to 377 in 2007. Samples of *A. hyperantus* ranged from 623 individuals in 1977 to 111 994 in 2009; the number of sites ranged from 25 in 1977 to 740 in 2009. Samples of *P. tithonus* ranged from 6845 individuals counted in 1978 to 107 994 in 2004; the number of sites ranged from 33 in 1976 to 647 in 2008. Samples of *L. megera* ranged from 484 individuals counted in 1977 to 4600 in 1990; the number of sites ranged from 33 in 1976 to 187 in 2004. *Lasiommata megera* is in decline in the U.K. (Asher *et al.*, 2011), which explains why the maximum count occurred relatively early in the time series, although the number of survey sites increased after 1990. Samples of *P. icarus* ranged from 1610 individuals counted in 1977 to 65 165 in 2003; the number of sites ranged from 35 in 1976 to 781 in 2009.

**Fig. 3:**
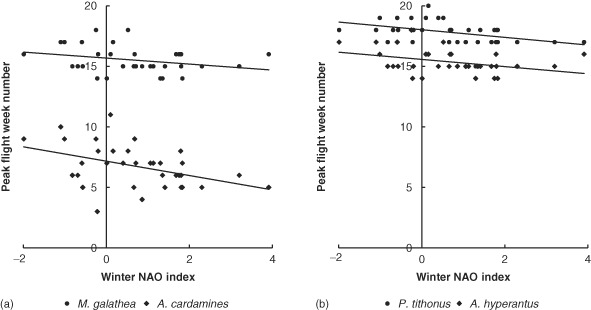
Winter North Atlantic Oscillation (NAO) indices and flight phenology for the four univoltine species. (a) Relationships between peak flight weeks of *Melanargia galathea* (*r* = −0.284, *P* = 0.103) and *Anthocharis cardamines* (*r* = −0.429, *P* = 0.011) and winter NAO index. (b) Relationships between peak flight weeks of *Pyronia tithonus* (*r* = −0.424, *P* = 0.012) and *Aphantopus hyperantus* (*r* = −0.375, *P* = 0.029) and winter NAO index. Each data point is the annual peak flight week number for 1976–2009.

**Fig. 4:**
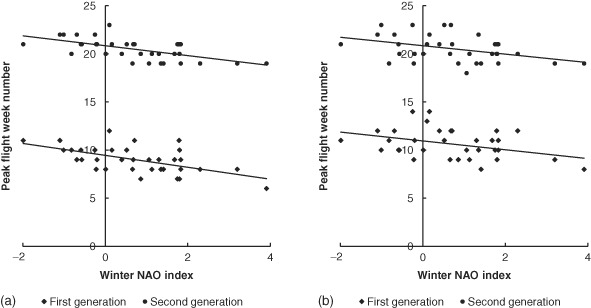
Winter North Atlantic Oscillation (NAO) indices and flight phenology for the two bivoltine species. (a) Relationships between the first (*r* = −0.577, *P*≤ 0.001) and second (*r* = −0.606, *P*≤ 0.001) generation peak flight weeks of *Lasiommata megera* and winter NAO index. (b) Relationships between the first (*r* = −0.382, *P* = 0.026) and second (*r* = −0.405, *P* = 0.018) generation peak flight weeks of *Polyommatus icarus* and winter NAO index. Each data point is the annual peak flight week number for 1976–2009.

The peak flight time for all four univoltine species and both generations of the two bivoltine species occurred earlier in years with a more positive winter NAO index (Figs 3 and 4). The winter NAO is associated with variations in the peak flight week of: 3.50 weeks for *An. cardamines* (*r* = −0.429, *P* = 0.011); 1.46 weeks for *M. galathea* (*r* = −0.284, *P* = 0.103); 1.76 weeks for *A. hyperantus* (*r* = −0.375, *P* = 0.029); 1.86 weeks for *P. tithonus* (*r* = −0.424, *P* = 0.012); 3.66 weeks for the first generation of *L. megera* (*r* = −0.577, *P*≤ 0.001); 3.03 weeks for the second generation of *L. megera* (*r* = −0.606, *P*≤ 0.001); 2.72 weeks for the first generation of *P. icarus* (*r* = −0.382, *P* = 0.026); and 2.58 weeks for the second generation of *P. icarus* (*r* = −0.405, *P* = 0.018). Each of the correlation coefficients calculated between the peak flight week and the winter NAO index (Figs 3 and 4) showed stronger associations than the correlation coefficients between the annual collated indices and the winter NAO index (Table 2) for the same species.

**Fig. 5:**
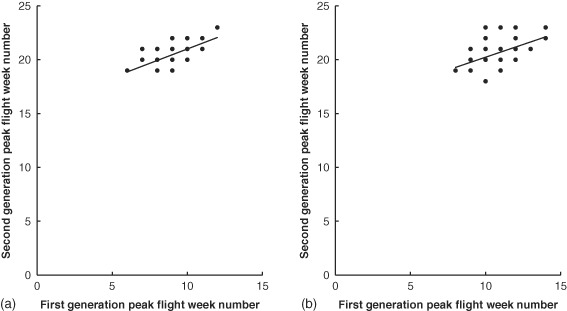
Second generation peak flight week number against first generation peak flight week number for (a) *Lasiommata megera* (*r* = 0.676, *P*≤ 0.001) and (b) *Polyommatus icarus* (*r* = 0.528, *P* = 0.001). Data are for all U.K. sites during 1976–2009. Each data point is for 1 year, but some data points are superimposed.

*Lasiommata megera* and *P. icarus* are bivoltine species in which the first generation flies nearer to the period during which weather is affected by the NAO than the second generation, yet both generations showed significant or highly significant correlations between the timing of peak flight weeks and the winter NAO index. Therefore, the association between the timing of the first generation and the timing of the second generation was analysed (Fig. 5). This identified highly significant Pearson correlations between the timing of the first and second generations of *L. megera* (*r* = 0.676, *P*≤ 0.001) and *P. icarus* (*r* = 0.528, *P* = 0.001). *Lasiommata megera* and *P. icarus* path coefficients for the Model 1 direct path (winter NAO index → first generation peak flight week → second generation peak flight week) were higher than for the Model 2 direct and indirect paths (winter NAO index → first generation peak flight week → second generation peak flight week, and winter NAO index → second generation peak flight week), which suggests that the primary cause of the timing of the second generation of both species was the effect of weather associated with the winter NAO on the timing of the first generation. All path coefficients, variance estimates and *P*-values are shown in Table 3.

**Table 3:** Path analysis

	*Lasiommata megera*		*Polyommatus icarus*	
		
	Model 1	Model 2	Model 1	Model 2
*Path coefficients (SE, P-value)*				
NAO index → peak 1	**−0.62**	**−0.62**	**−0.46**	**−0.46**
	(0.15; <0.01)	(0.15; <0.01)	(0.19; 0.017)	(0.19; 0.017)
Peak 1 → peak 2	**0.53**	**0.39**	**0.47**	**0.39**
	(0.10; <0.01)	(0.12; <0.01)	(0.13; <0.01)	(0.14; <0.01)
NAO index → peak 2	—	**−0.27**	—	−0.26
		(0.12; 0.028)		(0.17; 0.12)
*Variance estimates (SE)*				
NAO index	1.63 (0.40)	1.63 (0.40)	1.63 (0.40)	1.63 (0.40)
Peak 1	1.25 (0.31)	1.25 (0.31)	2.02 (0.50)	2.02 (0.50)
Peak 2	0.63 (0.16)	0.55 (0.14)	1.36 (0.34)	1.27 (0.31)

Model 1: winter NAO index → peak 1 → peak 2; Model 2: winter NAO index → peak 1 → peak 2, and winter NAO index → peak 2. SE, standard error; NAO, North Atlantic Oscillation; peak 1, first generation peak flight week number; peak 2, second generation peak flight week number. Path coefficients in bold are statistically significant at the 5% level.

The winter NAO index was a mean for a 3-month period because it was calculated from the January–March NAO indices. Therefore, multiple linear regression analysis was used to increase the resolution to specific months when weather influenced phenology. Overall model *r*^2^-values were higher for monthly temperatures than for monthly precipitation. This analysis showed significant and highly significant negative regressions between peak flight weeks and mean monthly temperatures for *An. cardamines* in April, *M. galathea* and *A. hyperantus* in April, May and June, and *P. tithonus* in June and July (Table 4). The peak flight week for first-generation *L. megera* showed negative regressions with *P*≤ 0.100 for all 4 months between February and May, and that for second-generation *L. megera* showed a significant negative regression with May temperature. The peak flight week for first-generation *P. icarus* showed a significant regression with June and July temperatures (Table 5).

**Table 4:** Multiple linear regression analyses between mean monthly temperatures, monthly precipitation and peak flight week for the univoltine species *Anthocharis cardamines*, *Melanargia galathea*, *Aphantopus hyperantus* and *Pyronia tithonus*

		Temperature			Precipitation		
					
		*r*^2^	*b*	*P*-value	*r*^2^	*b*	*P*-value
*An. cardamines*	October	0.715	0.113	0.451	0.371	−0.015	0.077
	November		0.151	0.429		0.003	0.727
	December		−0.095	0.505		−0.003	0.764
	January		−0.169	0.132		0.002	0.833
	February		−0.156	0.343		0.006	0.574
	March		−0.189	0.325		0.031	0.004**
	April		−0.941	<0.001**		0.010	0.315
	May		−0.204	0.439		0.004	0.674
*M. galathea*	October	0.726	0.256	0.017*	0.384	−0.001	0.854
	November		0.124	0.358		0.002	0.802
	December		0.025	0.794		0.004	0.583
	January		−0.058	0.479		<0.001	0.939
	February		0.054	0.674		0.016	0.033*
	March		0.208	0.153		0.010	0.130
	April		−0.434	0.004**		0.005	0.420
	May		−0.412	0.050*		0.002	0.763
	June		−0.320	0.037*		0.005	0.432
	July		0.012	0.934		−0.007	0.307
	August		−0.157	0.337		−0.001	0.903
*A. hyperantus*	October	0.744	0.231	0.015	0.405	−0.004	0.451
	November		0.022	0.854		0.002	0.688
	December		−0.052	0.538		0.001	0.925
	January		0.033	0.648		0.005	0.371
	February		0.033	0.774		0.011	0.100
	March		−0.055	0.663		0.012	0.065
	April		−0.375	0.005**		−0.004	0.526
	May		−0.382	0.041*		0.002	0.736
	June		−0.322	0.019*		0.005	0.372
	July		−0.057	0.666		−0.011	0.075
	August		−0.050	0.727		0.001	0.847
*P. tithonus*	October	0.734	0.136	0.120	0.298	0.001	0.850
	November		0.062	0.585		−0.001	0.857
	December		0.065	0.427		0.002	0.761
	January		−0.069	0.319		0.003	0.598
	February		−0.077	0.482		0.005	0.475
	March		0.152	0.215		0.005	0.398
	April		−0.099	0.394		0.006	0.330
	May		−0.163	0.343		−0.002	0.764
	June		−0.270	0.036*		0.008	0.153
	July		−0.343	0.011*		0.007	0.276
	August		−0.028	0.836		0.004	0.457

* *P* < 0.05

** *P* < 0.01.

*r*^2^, overall model coefficient of determination; *b*, regression coefficient.

**Table 5:** Multiple linear regression analyses between mean monthly temperatures, monthly precipitation and peak flight week for each of the two generations for the bivoltine species *Lasiommata megera* and *Polyommatus icarus*

		Temperature			Precipitation		
					
		*r*^2^	*b*	*P*-value	*r*^2^	*b*	*P*-value
*L. megera*, first generation	October	0.786	0.061	0.561	0.246	−0.007	0.315
	November		−0.178	0.183		0.002	0.825
	December		−0.093	0.351		0.002	0.761
	January		−0.059	0.459		−0.003	0.682
	February		−0.207	0.077		−0.008	0.375
	March		−0.269	0.062		0.012	0.159
	April		−0.283	0.054		−0.009	0.292
	May		−0.462	0.025*		0.007	0.462
	June		0.045	0.762		−0.003	0.655
*L. megera*, second generation	October	0.743	−0.036	0.724	0.312	−0.002	0.797
	November		−0.031	0.814		0.003	0.680
	December		0.060	0.518		0.006	0.415
	January		−0.209	0.014*		−0.005	0.416
	February		−0.082	0.516		−0.006	0.405
	March		0.037	0.790		0.003	0.657
	April		−0.149	0.273		0.004	0.531
	May		−0.484	0.024*		0.005	0.498
	June		0.006	0.968		−0.004	0.511
	July		−0.182	0.222		−0.004	0.630
	August		0.013	0.934		−0.001	0.851
	September		0.109	0.471		−0.013	0.063
*P. icarus*, first generation	October	0.573	−0.193	0.249	0.297	<0.001	0.970
	November		0.035	0.867		−0.001	0.887
	December		−0.042	0.789		0.005	0.582
	January		−0.022	0.862		0.001	0.866
	February		−0.221	0.224		−0.003	0.777
	March		−0.210	0.347		0.018	0.059
	April		−0.422	0.068		−0.002	0.808
	May		−0.287	0.358		−0.004	0.715
	June		−0.316	0.185		−0.015	0.080
*P. icarus*, second generation	October	0.784	0.012	0.920	0.225	−0.009	0.263
	November		0.069	0.657		0.007	0.467
	December		0.057	0.604		−0.006	0.526
	January		0.108	0.246		0.007	0.375
	February		−0.218	0.145		0.011	0.313
	March		−0.176	0.283		0.019	0.065
	April		−0.290	0.074		0.010	0.323
	May		−0.120	0.612		0.005	0.638
	June		−0.700	<0.001**		0.003	0.755
	July		−0.472	0.011*		−0.001	0.917
	August		0.031	0.865		<0.001	0.958
	September		0.119	0.500		−0.003	0.710

* *P* < 0.05,

** *P* < 0.01.

*r*^2^, overall model coefficient of determination; *b* = regression coefficient.

There was a highly significant positive association between March precipitation and peak flight week in *An. cardamines* and a significant positive association between February precipitation and peak flight week in *M. galathea*. There were no significant regressions between monthly precipitation and peak flight week for *A. hyperantus*, *P. tithonus* or first- or second-generation *L. megera* and *P. icarus* (Tables 4 and 5).

## Discussion

### The NAO, climate change and weather

The identification of when the NAO has the greatest effect on temperature and precipitation was prerequisite to describing how the NAO influences butterfly populations. The NAO influenced temperatures from October to April, and the strongest association was identified between December and February. The NAO had greater influence over temperature than precipitation, but showed significant influence over precipitation in January and February (Table 1).

Mean annual temperature in the U.K. increased by 1.22 °C during the period 1976–2009 (Fig. 2) and butterflies have emerged earlier in response to recent climate warming (Sparks & Yates, 1997; Roy & Sparks, 2000). Although there was a highly significant correlation between the winter NAO index and mean annual temperature (*r* = 0.544, *P* = 0.001), the winter NAO index did not show an upward trend during the time series and thus would appear not to have made a major contribution to the overall temperature increase. The NAO is an oscillatory set of weather parameters and is different from climate change. Conclusions as to whether there is an association between rises in temperatures caused by climate change and the NAO can be influenced by the time window studied, particularly in shorter time series. It is likely that climate change and the NAO combine to influence the phenology of butterfly flight, although Wallisdevries and Van Swaay (2006) hypothesise that climate warming combined with high nitrogen deposition can advance spring plant growth, leading to microclimatic cooling, which can affect butterfly species that hibernate as eggs or larvae. Such suggestions indicate that the relationship between weather and butterflies can be complex.

### The role of the NAO in butterfly abundance

The multi-species collated index did not show an association with the winter NAO index. However, multi-species indices are averages of the response of many species to a range of environmental factors, and hence may mask effects on some individual species, and thus Pearson correlation coefficients were calculated to investigate the relationships between the annual collated indices for each of 35 individual species and the winter NAO index (Table 2). A significant relationship was found for only one species (*L. megera*), but bivoltine species tended to have stronger associations with the winter NAO index than univoltine species. Similarly, population abundances of species with relatively long flight periods tended to be more positively associated with the NAO index.

An explanation for the finding of positive associations between bivoltine species and the winter NAO index is that warmer weather, associated with a positive NAO index, provided a longer time in which to complete two or more generations. Altermatt (2010) has shown that climatic warming increases voltinism in European butterflies and moths, and Välimäki *et al.* (2008) have discussed the issues of seasonal weather, time constraint and number of generations. The low correlations with univoltine species may imply that as their flight seasons are shorter, weather is less important in terms of allowing sufficient time to complete this life stage and the negative associations shown by some univoltine species suggest these species require cold winters, perhaps for dormancy. It is possible that the winter NAO index can be used to predict the relative abundance of univoltine and bivoltine species, and it is also possible that increased temperatures associated with climate change may favour bivoltine species more than univoltine species. Therefore, the NAO appears to have a complex relationship with butterfly lifecycle parameters and abundance, which was difficult to detect using multi-species indicators. As the NAO plays such an important role in U.K. weather, it is perhaps questionable how suitable multi-species indices are for monitoring ecological change. There may be an important, and difficult, practical dilemma to develop an index of entomological population change that integrates complex ecological traits and can identify conservation problems. I suspect you don't like ‘practical dilemma’? Is there an alternative word such as challenge.

### The role of the NAO in butterfly phenology

The NAO influenced the timing, or phenology, of the butterfly flight season for all six species studied in detail. The peak flight weeks for *An. cardamines*, *M. galathea*, *A. hyperantus* and *P. tithonus* (Fig. 3), and the peak flight weeks for both the first and second generations of *L. megera* and *P. icarus* (Fig. 4) were earlier in positive NAO index years. These results were similar to those found for the green spruce aphid, *E. abietinum*, which flew earlier in years with a more positive winter NAO index (Westgarth-Smith *et al.*, 2007). The fact that correlation coefficients between peak flight week and the winter NAO index were higher than those between annual collated indices and the winter NAO index indicated that the NAO had a stronger relationship with butterfly phenology than with abundance. The NAO index is very difficult to predict and hence although the it can be used to predict flight timing, it would be difficult to do so in December, but feasible to do so by the end of March because by then the winter NAO index is known.

Temperature had a greater effect on flight timing than precipitation. Warmer temperatures during the period April–July resulted in the earlier flight of all six species of butterfly; temperatures in later months are associated with later flying species. Previous studies (Sparks & Yates, 1997; Roy & Sparks, 2000) have also found that warmer spring weather is associated with earlier butterfly flight timing. Sparks and Yates (1997) showed how the flights of four U.K. butterfly species occurred earlier in years when temperatures in April were higher. The present study also suggests that higher precipitation in February and March can delay the flight timing of two of the univoltine species. The NAO index is significantly positively correlated with both temperature and precipitation in February (Table 1) and thus to some extent the effect of higher temperature on flight timing (making a flight season earlier) should be partly offset by the effect of higher precipitation (making the flight season later). However, as temperatures were more strongly correlated with the NAO index for more months than precipitation, the mechanism by which the NAO exerts control on flight timing probably refers mainly to temperature. Thus, although other authors have shown associations between weather in specific months and flight timing, our new contribution links these associations to the NAO.

It appears that a factor determining the timing of the second generation in bivoltine species is the timing of the first generation; this presumably reflects the length of time needed for eggs laid in the first generation to hatch, develop as larvae, pupate and emerge as adults, rather than weather conditions at the time of, or shortly before, the emergence of the second generation as adults. Effectively, the NAO was found to indirectly control the phenology of the second generation by controlling the phenology of the first generation. We believe that this is the first time an association between the timing of the first and second generations in bivoltine species has been described using butterfly ecological datasets.

In conclusion, the use of multi-species indicators hides the complexity of response in individual species. Butterfly species that are bivoltine and have longer flight periods were found to be more likely to respond positively to the NAO index than univoltine species with short flight periods. Warmer weather associated with a more positive winter NAO index caused butterflies to fly earlier. The NAO controls temperature, which, in bivoltine species, controls the timing of the first generation; subsequently, the timing of the first generation controls the timing of the second generation.
